# Supplementary figures and images for: Diversity and inclusion: A hidden additional benefit of Open Data
Source: PLOS Digit Health. 2024 Jul 23;3(7):e0000486. doi: 10.1371/journal.pdig.0000486 (PMC11265679; doi:10.1371/journal.pdig.0000486)

**Supplementary Figure 1. Distribution of the top 10 venues among papers in the treatment group.**


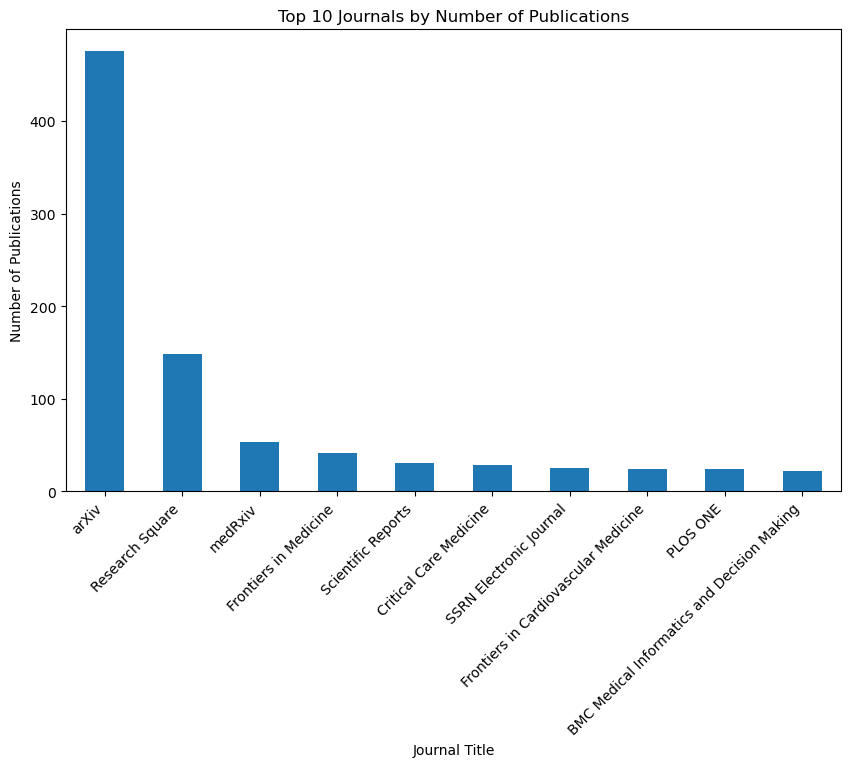

Supplement: S1 Fig — (DOCX) [file pdig.0000486.s001.docx]

**Supplementary Figure 2.** Distribution of the top 10 venues among papers in the control group.


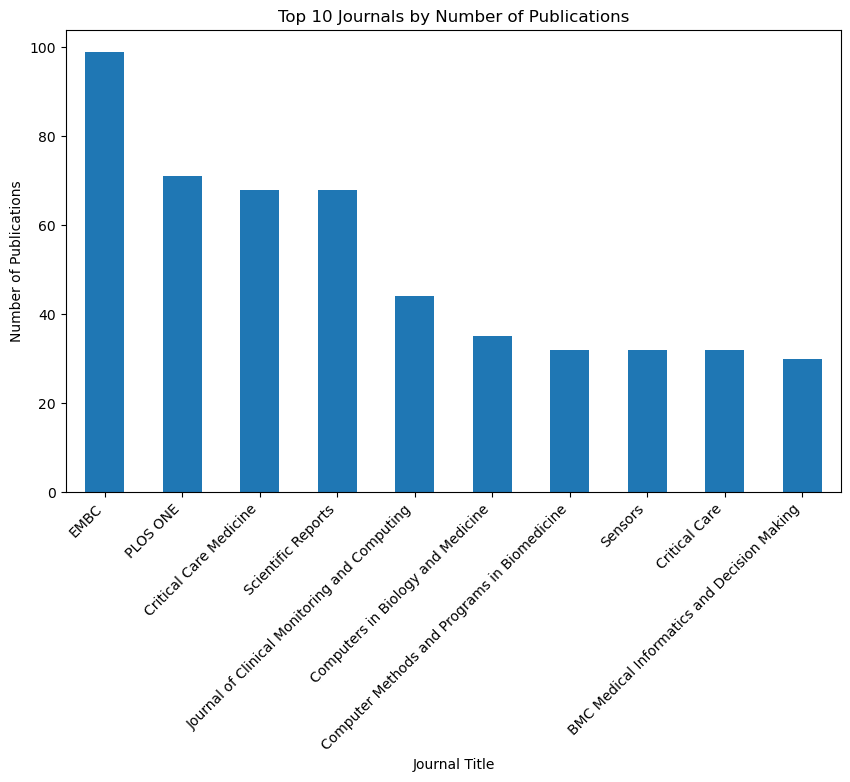

Supplement: S2 Fig — (DOCX) [file pdig.0000486.s002.docx]
