# Supplementary material for: Diversity and inclusion: A hidden additional benefit of Open Data
Source: PLOS Digit Health. 2024 Jul 23;3(7):e0000486. doi: 10.1371/journal.pdig.0000486 (PMC11265679; doi:10.1371/journal.pdig.0000486)
Supplement: S1 Table — (DOCX) [file pdig.0000486.s003.docx]

**Supplementary Table 1.** Results of the sensitivity analysis performed under the assumption that all authors with missing gender labels are women.

| **Role** | **Adjusted Treatment Count** | **Adjusted Treatment Proportion (%)** | **Adjusted Control Count** | **Adjusted Control Proportion (%)** | **Z-Statistic** | **P-Value** |
| --- | --- | --- | --- | --- | --- | --- |
| Woman first author | 909 | 39.4% | 1218 | 41.8% | -1.771 | 0.96169 |
| Woman last author | 812 | 35.2% | 1000 | 34.3% | 0.646 | 0.25930 |
