# Supplementary material for: Diversity and inclusion: A hidden additional benefit of Open Data
Source: PLOS Digit Health. 2024 Jul 23;3(7):e0000486. doi: 10.1371/journal.pdig.0000486 (PMC11265679; doi:10.1371/journal.pdig.0000486)
Supplement: S2 Table — (DOCX) [file pdig.0000486.s004.docx]

**Supplementary Table 2.** Results of the sensitivity analysis performed under the assumption that all authors with missing gender labels are men.

| **Role** | **Adjusted Treatment Count** | **Adjusted Treatment Proportion (%)** | **Adjusted Control Count** | **Adjusted Control Proportion (%)** | **Z-Statistic** | **P-Value** |
| --- | --- | --- | --- | --- | --- | --- |
| Woman first author | 541 | 27.9% | 755 | 30.8% | -2.111 | 0.98263 |
| Woman last author | 453 | 23.3% | 518 | 21.3% | 1.534 | 0.06255 |
