# Supplementary material for: Diversity and inclusion: A hidden additional benefit of Open Data
Source: PLOS Digit Health. 2024 Jul 23;3(7):e0000486. doi: 10.1371/journal.pdig.0000486 (PMC11265679; doi:10.1371/journal.pdig.0000486)
Supplement: S3 Table — (DOCX) [file pdig.0000486.s005.docx]

# **Supplementary Table 3.** Results of the sensitivity analysis using the method of imputing missing data from the distribution of authors with gender labels.

| **Role** | **Adjusted Treatment Count** | **Adjusted Treatment Proportion (%)** | **Adjusted Control Count** | **Adjusted Control Proportion (%)** | **Z-Statistic** | **P-Value** |
| --- | --- | --- | --- | --- | --- | --- |
| Woman first author | 643 | 27.9% | 897 | 30.8% | -2.306 | 0.98946 |
| Woman last author | 536 | 23.2% | 620 | 21.3% | 1.678 | 0.04665 |
