# Supplementary material for: Diversity and inclusion: A hidden additional benefit of Open Data
Source: PLOS Digit Health. 2024 Jul 23;3(7):e0000486. doi: 10.1371/journal.pdig.0000486 (PMC11265679; doi:10.1371/journal.pdig.0000486)
Supplement: S4 Table — (DOCX) [file pdig.0000486.s006.docx]

# **Supplementary Table 4.** Results of the sensitivity analysis using the method of imputing missing data from the distribution of authors with labels (LMIC vs. not LMIC).

| **Role** | **Adjusted Treatment Count** | **Adjusted Treatment Proportion (%)** | **Adjusted Control Count** | **Adjusted Control Proportion (%)** | **Z-Statistic** | **P-Value** |
| --- | --- | --- | --- | --- | --- | --- |
| LMIC author in any position | 232 | 10.1 | 192 | 8.3 | 4.55 | <0.001 |
| LMIC author in first position | 166 | 7.2 | 123 | 5.3 | 4.66 | <0.001 |
| LMIC author in last position | 164 | 7.1 | 130 | 5.6 | 4.12 | <0.001 |
