# Supplementary material for: Diversity and inclusion: A hidden additional benefit of Open Data
Source: PLOS Digit Health. 2024 Jul 23;3(7):e0000486. doi: 10.1371/journal.pdig.0000486 (PMC11265679; doi:10.1371/journal.pdig.0000486)
Supplement: S5 Table — (DOCX) [file pdig.0000486.s007.docx]

**Supplementary Table 5.** Results of the sensitivity analysis performed under the assumption that all missing authors are from a LMIC.

| **Role** | **Adjusted Treatment Count** | **Adjusted Treatment Proportion (%)** | **Adjusted Control Count** | **Adjusted Control Proportion (%)** | **Z-Statistic** | **P-Value** |
| --- | --- | --- | --- | --- | --- | --- |
| LMIC author in any position | 970 | 42.0 | 386 | 16.7 | 23.55 | <0.001 |
| LMIC author in first position | 927 | 40.2 | 332 | 14.4 | 24.14 | <0.001 |
| LMIC author in last position | 926 | 40.1 | 339 | 14.7 | 23.86 | <0.001 |
