# Supplementary material for: Diversity and inclusion: A hidden additional benefit of Open Data
Source: PLOS Digit Health. 2024 Jul 23;3(7):e0000486. doi: 10.1371/journal.pdig.0000486 (PMC11265679; doi:10.1371/journal.pdig.0000486)
Supplement: S6 Table — (DOCX) [file pdig.0000486.s008.docx]

**Supplementary Table 6.** Results of the sensitivity analysis performed under the assumption that all missing authors are not from a LMIC.

| **Role** | **Adjusted Treatment Count** | **Adjusted Treatment Proportion (%)** | **Adjusted Control Count** | **Adjusted Control Proportion (%)** | **Z-Statistic** | **P-Value** |
| --- | --- | --- | --- | --- | --- | --- |
| LMIC author in any position | 150 | 6.5 | 168 | 7.3 | 1.099 | 0.13589 |
| LMIC author in first position | 107 | 4.6 | 114 | 4.9 | 1.289 | 0.09878 |
| LMIC author in last position | 106 | 4.6 | 121 | 5.2 | 0.773 | 0.21974 |
