# Supplementary material for: Diversity and inclusion: A hidden additional benefit of Open Data
Source: PLOS Digit Health. 2024 Jul 23;3(7):e0000486. doi: 10.1371/journal.pdig.0000486 (PMC11265679; doi:10.1371/journal.pdig.0000486)
Supplement: S1 Text — (DOCX) [file pdig.0000486.s009.docx]

**Supplementary Text 1.** **Brief overview of the four open-access critical care databases considered in our study.**

Herein, we provide more details about the nature of the open critical care databases considered in our study.

For the treatment group, we considered the two most popular open-access critical care databases: MIMIC (II, III, IV) and eICU. All MIMIC databases comprise patients hospitalized at a large academic hospital in the Boston area, thus primarily capturing US patients from the New England region receiving acute care. In contrast, the eICU database comprises patients who were admitted to one of 335 units at 208 hospitals located throughout the US and is therefore more representative of the country as a whole rather than specific to a particular geographical region. Below, we provide more specific information about each of MIMIC-II, MIMIC-III, and MIMIC-IV as well as eICU.

- MIMIC-II: The database includes over 26,000 adult hospital admissions and over 31,000 adult ICU stays that occurred between 2001 and 2008. The median age at admission was 65.5. Over 56% of the patients were male. In this population, in-hospital mortality was 11.5%. More details are available in Table 2 ([Lee et al., Conf Proc IEEE Eng Med Biol Soc, 2011](https://www.ncbi.nlm.nih.gov/pmc/articles/PMC6339457/)).
- MIMIC-III: As explained by the authors ([Johnson et al., Physionet, September 2016](https://physionet.org/content/mimiciii/1.4/)): *“MIMIC-III is a large, freely-available database comprising de-identified health-related data associated with over forty thousand patients who stayed in critical care units of the Beth Israel Deaconess Medical Center between 2001 and 2012.”* Notably, MIMIC-III is an extension of MIMIC-II (adding 2008-2012), resulting in a large overlap between MIMIC-II and MIMIC-III. A more detailed characterization of the underlying patient population is provided in Table 1 ([Johnson et al., Scientific Data, May 2016](https://www.nature.com/articles/sdata201635)), stratified by the first critical care unit on hospital admission for patients aged 16 years and above. Overall, the dataset includes 38,597 distinct adult patients and 49,785 hospital admissions. The median age of adult patients was 65.8 years. Moreover, 55.9% were male patients. In that population, in-hospital mortality was 11.5%. These summary statistics align with those from MIMIC-II.
- MIMIC-IV: The MIMIC-IV database was released more recently, in 2023 ([Johnson et al., Scientific Data, January 2023](https://www.nature.com/articles/s41597-022-01899-x)). Importantly, it includes not only ICU patients but also hospitalized patients who were not admitted or transferred to an ICU. It is much larger than MIMIC-III, with a total of 180,000+ unique hospitalized patients, including 50,000+ who received ICU care. Among hospitalized (ICU) patients, the median age was 58.8 (64.7) and the proportion of men was 47.8% (55.8%). In-hospital mortality was 2.1% (resp. 11.6%). More details are available in Table 1 of the aforementioned paper.
- eICU: The eICU database was released after MIMIC-II and MIMIC-III, but before MIMIC-IV ([Pollard et al., Scientific Data, September 2018](https://www.nature.com/articles/sdata2018178)). It includes over 139,000 unique patients admitted between 2014 and 2015. The median age at admission was 65; 54% of the patients were male. For ICU patients, in-hospital mortality was lower than in MIMIC databases, at 9.0%. More details are available in Table 1 of the aforementioned paper.
