# Supplementary material for: Diversity and inclusion: A hidden additional benefit of Open Data
Source: PLOS Digit Health. 2024 Jul 23;3(7):e0000486. doi: 10.1371/journal.pdig.0000486 (PMC11265679; doi:10.1371/journal.pdig.0000486)
Supplement: S2 Text — (DOCX) [file pdig.0000486.s010.docx]

**Supplementary Text 2. Further details about our assumptions underlying the statistical tests used in the study.**

We used the Chi-Squared test of independence to determine whether there was a relationship between an author’s gender and their propensity to have a preprint or published paper leveraging an open-access rather than a private critical care database. Beyond gender, we tested similar hypotheses with respect to the LMIC status of the country of an author’s affiliated institution and the MSI status of their institution. Note that these three variables (i.e., gender, LMIC status, and MSI status) are all categorical variables, measured at an ordinal or nominal level.

In order to apply the Chi-Squared test of independence:

1. First, three key assumptions need to be satisfied:
   1. Analyzing a representative sample from a superpopulation;
   2. Comparing mutually exclusive groups and categories;
   3. Having independent units.
2. Second, common practice is to clearly state the hypotheses being tested.
3. Third, a minimum sample size is required for each cell (e.g., women in the control group). Below, we provide a justification for each of these elements.

Requirement #1: Three key assumptions

First, we comment on the validity of the three key assumptions listed above.

***a.*** *Representative sample of papers*

Given our inclusion criteria, based on a well-performing model to identify AI-related publications and query search terms specific to critical care curated by Van de Sande et al. and vetted by two physician authors on our team, we expect that the sample of papers we collected is representative of the population of papers on artificial intelligence in critical care, resulting in a representative sample from the population of authors writing on this topic.

***b.*** *Mutually exclusive groups*

Importantly, we ensured that no paper was simultaneously in the treatment and control group. This step helped confirm that the assumption of mutually exclusive groups was met for the results presented in **Table 1B**, where papers constituted the unit of analysis. Although certain authors may be present in both the treatment and control groups (because they wrote at least one paper about an open database and at least one paper about a private database), the prevalence of that scenario was rare. Therefore, the extent to which the assumption that groups are mutually exclusive is violated is quite limited for the results presented in **Table 1A**.

***c.*** *Independence among units present in a given group*

When using papers as units, the independence assumption is generally satisfied, with the rare exception of a single team publishing two closely related papers (e.g., using the same methodology but applied to two different datasets) within a few days of one another. The situation is more complex when using authors as units. Although an author may contribute to several papers present in the treatment or control group, we removed duplicated author instances to ensure that each author appeared only once in their respective group. Therefore, units were not repeated. Although we built such groups of individual-level author units by disaggregating teams of authors (i.e., paper-level connections exist among them), we reasoned that each author made the decision to participate in the team and write the paper on an individual basis. Thus, we believe the potential for interference among authors (e.g., owing to homophily by gender) is limited, satisfying the assumption of independence among units.

In addition, we now state explicitly the set of hypotheses that we tested in Requirement #2 below.

Requirement #2: Hypothesis formulation

With respect to gender, we tested three hypotheses, each corresponding to a certain level of involvement: (i) serving as any author, (ii) serving as a first author, or (iii) serving as a last author. We similarly tested three hypotheses with respect to LMIC status and three hypotheses with respect to MSI status.

Null hypothesis: *There is not a relationship between an author’s gender (respectively, the MSI status of their affiliated institution or LMIC status of the corresponding country) and their propensity to have a preprint or published paper leveraging an open-access rather than a private critical care database (i.e., the two considered variables are independent).*

Alternative hypothesis: *There is a relationship between the two considered variables (i.e., they are dependent).*

Requirement #3: Minimum sample size per cell

After building contingency tables, we checked that all had cells with a number of units larger than 10 (i.e., the minimum generally required to run a Chi-Squared test of independence).

Furthermore, we applied a Bonferroni correction to handle multiple hypothesis testing. In total, we performed 19 tests. Upon application of the correction, all results reported with a p-value < 0.001 in the manuscript remained statistically significant.
